# Impact of aetiological screening of sexually transmitted infections during pregnancy on pregnancy outcomes in South Africa

**DOI:** 10.1186/s12884-022-04520-6

**Published:** 2022-03-09

**Authors:** Dorothy C. Nyemba, Remco P. H. Peters, Andrew Medina-Marino, Jeffrey D. Klausner, Phuti Ngwepe, Landon Myer, Leigh F. Johnson, Dvora L. Joseph Davey

**Affiliations:** 1 Division of Epidemiology & Biostatistics, School of Public Health and Family Medicine, University of Cape Town, Cape Town, South Africa; 2 Centre for Infectious Disease Epidemiology and Research, School of Public Health and Family Medicine, University of Cape Town, Cape Town, South Africa; 3 Research Unit, Foundation for Professional Development, East London, South Africa; 4 Department of Medical Microbiology, University of Pretoria, Pretoria, South Africa; 5 Division of Medical Microbiology, University of Cape Town, Cape Town, South Africa; 6 Desmond Tutu HIV Centre, University of Cape Town, Cape Town, South Africa; 7 Department of Psychiatry, Perelman School of Medicine, University of Pennsylvania, Philadelphia, USA; 8 Department of Population and Public Health Sciences, University of Southern California, Keck School of Medicine, Los Angeles, USA; 9 Department of Preventive Medicine, Epidemiology, Fielding School of Public Health, University of California Los Angeles, Los Angeles, USA

## Abstract

**Background:**

Sexually transmitted infections (STIs) during pregnancy may increase the risk of adverse pregnancy outcomes. STI syndromic management is standard of care in South Africa but has its limitations. We evaluated the impact of diagnosing and treating curable STIs during pregnancy on adverse pregnancy and birth outcomes.

**Methods:**

We combined data from two prospective studies of pregnant women attending public sector antenatal care (ANC) clinics in Tshwane District and Cape Town, South Africa. Pregnant women were enrolled, tested and treated for STIs. We evaluated the association between any STI at the first ANC visit and a composite adverse pregnancy outcome (miscarriage, stillbirth, preterm birth, early neonatal death, or low birthweight) using modified Poisson regression models, stratifying by HIV infection and adjusting for maternal characteristics.

**Results:**

Among 619 women, 61% (*n* = 380) were from Tshwane District and 39% (*n* = 239) from Cape Town; 79% (*n* = 486) were women living with HIV. The prevalence of any STI was 37% (*n* = 228); *C. trachomatis,* 26% (*n* = 158), *T. vaginalis,* 18% (*n* = 120) and *N. gonorrhoeae*, 6% (*n* = 40). There were 93% (*n* = 574) singleton live births, 5% (*n* = 29) miscarriages and 2% (*n* = 16) stillbirths. Among the live births, there were 1% (*n* = 3) neonatal deaths, 7% (*n* = 35) low birthweight in full-term babies and 10% (*n* = 62) preterm delivery. There were 24% (*n* = 146) for the composite adverse pregnancy outcome. Overall, any STI diagnosis and treatment at first ANC visit was not associated with adverse outcomes in women living with HIV (adjusted relative risk (aRR); 1.43, 95% CI: 0.95–2.16) or women without HIV (aRR; 2.11, 95% CI: 0.89–5.01). However, *C. trachomatis* (aRR; 1.57, 95% CI: 1.04–2.39) and *N. gonorrhoeae* (aRR; 1.69, 95% CI: 1.09–3.08), were each independently associated with the composite adverse outcome in women living with HIV.

**Conclusion:**

Treated STIs at the first ANC visit were not associated with adverse pregnancy outcome overall. In women living with HIV, *C. trachomatis* or *N. gonorrhoeae* at first ANC were each independently associated with adverse pregnancy outcome. Our results highlights complex interactions between the timing of STI detection and treatment, HIV infection and pregnancy outcomes, which warrants further investigation.

**Supplementary Information:**

The online version contains supplementary material available at 10.1186/s12884-022-04520-6.

## Background

Curable sexually transmitted infections (STIs), specifically *C. trachomatis, T. vaginalis* and *N. gonorrhoeae* during pregnancy have been associated with several adverse pregnancy and birth outcomes. Those adverse outcomes include miscarriage, stillbirth, prematurity, low birth weight and several secondary life-threatening conditions in surviving neonates [1–4]. However, there is limited consensus on the benefit of diagnosis and treatment of STIs, outside of syphilis, during pregnancy and reducing adverse pregnant outcomes. In pregnant women, untreated *C. trachomatis* infection has been associated with adverse obstetric outcomes such as fetal loss, premature rupture of membranes, preterm labour and preterm delivery in most but not all studies [5–8]. Maternal *C. trachomatis* infections can be transmitted during delivery and lead to conjunctivitis and pneumonia in newborns [5, 9, 10]. Maternal *N. gonorrhoeae* infection is associated with adverse pregnancy outcomes such as low birthweight [11, 12], small for gestational age (SGA) [1], preterm delivery, premature rupture of membranes and septic abortion. Newborns of mothers with *N. gonorrhoeae* infection can contract neonatal conjunctivitis which can lead to blindness if left untreated [13]. Maternal *T. vaginalis* infection can lead to low birthweight, preterm delivery, premature rupture of membranes, mental retardation, and vaginal and respiratory infections in neonates [14–16].

Despite the major threat to maternal and child health posed by these curable STIs, antenatal screening for *C. trachomatis, N. gonorrhoeae* and *T. vaginalis* infections is not recommended by WHO nor is part of the routine standard of care in antenatal settings in most developing countries [17]. Although symptomatic cases may be screened and offered treatment, most of these curable STIs are asymptomatic and often go without diagnosis and treatment [18, 19]. Most developing countries, introduced universal screening of syphilis in pregnant women attending antenatal clinics since 2012 [20, 21]. However, this has not been the case with other curable STIs such as *C. trachomatis, N. gonorrhoeae* and *T. vaginalis* because of the lack of laboratory infrastructure and financial resources to support aetiological screening in pregnancy [18, 19, 22]. However, point-of-care molecular tests such as GeneXpert® for *C. trachomatis, N. gonorrhoeae and T. vaginalis* have demonstrated high acceptability [6], feasibility and reliability [23, 24] in low- and middle-income countries, and could be scaled up to support screening for asymptomatic STIs in pregnancy.

South Africa’s antenatal HIV prevalence is one of the highest globally, reported at approximately 30% in 2017 [25]. In a recent study of pregnant women in South Africa in 2020, the prevalence and incidence of STIs was shown to be higher among women living with HIV compared to women without HIV [26]. In this high prevalence setting, more than 95% of HIV-infected pregnant women receive treatment for HIV infection in pregnancy, resulting in many newborns who are exposed to both HIV and antiviral treatment [27–29]. Recent studies are demonstrating that in utero fetal exposure to HIV and ARVs may be associated with adverse birth outcomes such as preterm birth and low birthweight [27–31]. In settings with double burden of HIV and STIs, there is a greater need to evaluate the impact of curable STIs in pregnancy on the pregnancy and birth outcome. In this study, we evaluated the impact of screening and treating STIs at first antenatal care visit on the prevalence of adverse pregnancy and birth outcomes stratifying by HIV status.

## Methods

### Study design and setting

We combined data from two observational prospective studies of pregnant women attending public sector antenatal clinics (ANCs) in Tshwane District and Cape Town, South Africa. Study enrolment in Tshwane District occurred between June 2016 and October 2017, and this study enrolled only women living with HIV. The study setting, eligibility criteria, data collection, and specimen collection and testing have been described elsewhere [19]. Study enrolment in Cape Town occurred between January 2018 and January 2019, and this study enrolled pregnant women living with and without HIV infection. Pregnant women with a confirmed HIV negative test result at enrolment, had repeat HIV testing at every antenatal appointment and after delivery. The study setting, eligibility criteria, data collection, and specimen collection and testing have been described elsewhere [18]. Briefly, in both studies pregnant women had to be: (i) ≥ 18 years of age, (ii) attending their first ANC visit and confirmed pregnancy using a urine pregnancy test, (iii) ≤ 35 weeks gestational age, (iv) confirmed HIV-infection status and (v) intenting to reside within the community for the duration of the pregnancy up to delivery.

### Specimen collection, testing and treatment

As previously described [18, 19], women were offered aetiological testing through vulvovaginal swab specimens using Xpert® Vaginal/Endocervical Specimen Collection kits (Cepheid, Sunnyvale, CA). Specimens were tested for *C. trachomatis, N. gonorrhoeae* and *T. vaginalis* at first ANC visit and the first visit postpartum within one month (4 weeks) postpartum. Specimens were tested at the same day, and most women (94%) were given results before leaving the clinic. All women with a positive STI test result received treatment on the same day based on the Xpert® result per the choice of drugs in line with the South African National guidelines [32]. As per the national STI guidelines, women were given counselling, and provided with condoms and partner notification/referral letter [26].

### Study definitions

For this analysis, pregnancy and obstetric outcomes were abstracted from obstetric records and maternity case records at delivery facilities. Birth anthropometrics were abstracted from the child Road to Health Booklets at the 7-day postpartum visit where mother-infant pairs were evaluated by study teams. Gestational age was estimated based on the date of the last menstrual period. Miscarriage was defined as noninduced pregnancy loss before 20 weeks of gestation. Stillbirth was defined as delivery of a baby with no sign of life at or after 20 weeks gestation. Early neonatal death was defined as death of a newborn between zero and seven days after birth. Preterm birth was defined as birth before completion of 37 weeks of gestation. Low birthweight was defined as an infant birthweight less than 2500 g. We used World Health Organization guidelines to categorize adverse birth outcomes [33]. The primary outcome of interest was a composite adverse outcome defined as any adverse pregnancy or birth outcome including miscarriage, stillbirth, early neonatal death, preterm birth or low birthweight. For sensitivity analysis, we excluded miscarriages (usually underestimated in outcomes of pregnancy cohorts due to left truncation as most women attend first ANC visit at gestational age after the typical period at which miscarriage occurs) in the composite adverse outcome. The exposure of interest was a positive diagnosis of a STI at first ANC visit. We also performed a sensitivity analysis by combining *C. trachomatis and N. gonorrhoeae* as exposure of interest excluding *T. vaginalis* with a different pathogenic inflammatory mechanism.

### Data management

Study data were collected and managed using Research Electronic Data Capture (REDCap) platform [34]. All study participants were allocated a unique participant identifier allocated at study enrolment and all electronic communications were done through password-protected and encrypted files.

### Statistical analysis

Maternal characteristics were compared using Wilcoxon test, χ2 test or Fisher exact test as appropriate. Odds Ratios are often used to approximate prevalence ratio or relative risk but can lead to overestimations for common events hence relative risk were used [35]. We fitted generalized linear models using modified Poisson regression with robust error measurements to estimate relative risk and 95% confidence interval (CI) for all association analyses. The modified Poisson regression with robust error measurements has fewer convergence problems compared to log-binomial logistic regression [35]. In stratified exploratory analysis by HIV status, we observed that women living with HIV were more likely to have composite adverse outcome compared to their counterpart women living without HIV, suggesting HIV status may be an effect modifier. Therefore, we performed several sensitivity analyses: first, exposure of interest as positive diagnosis of each STI (*C. trachomatis, N. gonorrhoeae* and *T. vaginalis)* to assess if a particular STI was associated with the composite adverse outcome. Second, we performed a sensitivity analyses with exposure of interest including *C. trachomatis and N. gonorrhoeae* only, third, with composite adverse outcome excluding miscarriages. Fourth, we used STIs at postnatal visit as proxy for STI status at time of delivery for women who had live births. All the models adjusted for maternal age, gestational age at enrolment, employment, marital status and gravidity.

### Ethical consideration

Ethical approvals were provided by the University of Cape Town’s Faculty of Health Sciences Research Ethics Committee (UCT-HREC, reference number 454/2017), University of Pretoria’s Faculty of Health Sciences Research Ethics Committee (reference number 401/2015) and the University of California Los Angeles (reference number 15–001351). All women participating in the two studies provided informed written consent for their respective study participation.

## Results

**Fig. 1:**
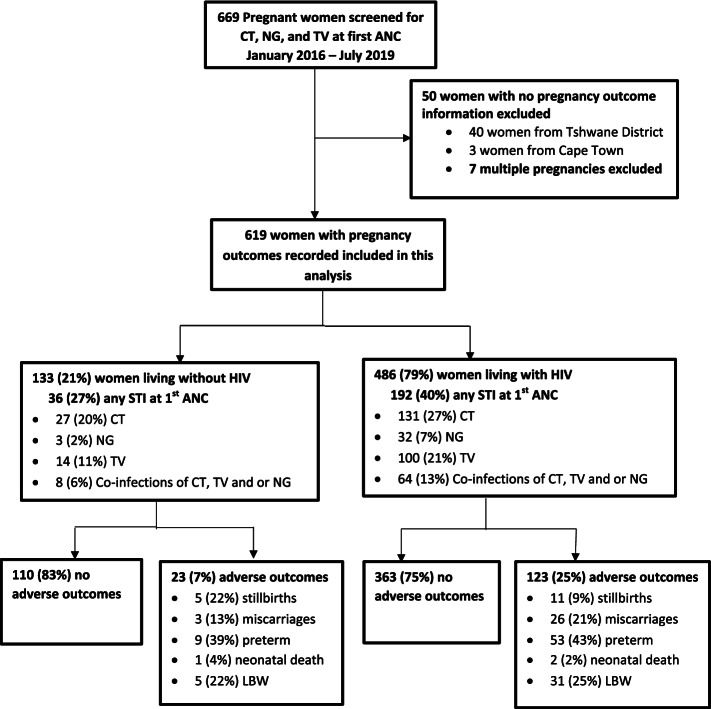
Flow chart of pregnancies and outcomes included in this analysis. CT, *Chlamydia trachomatis*; NG, *Neisseria gonorrhoeae*; TV, *Trichomonas vaginalis*; ANC, antenatal clinic. Preterm, gestational age < 37 weeks; miscarriage, gestational age ≤ 20 weeks; stillbirth, gestational age > 20 weeks, LBW, low birthweight < 2500 g and neonatal death, early neonatal death of new-born between 0 and 7 days after birth

A total of 669 pregnant women were enrolled at first ANC visit and followed until the first postpartum study visit. Women excluded from this analysis have reasons listed in Fig. 1. Of the 669 pregnant women, 93% (619) of the women who had pregnancy outcomes recorded are included in this data analysis, 61% (380) were from Tshwane District and 39% (239) from Cape Town. Overall, 79% (486) were women living with HIV and 21% (133) without HIV (Table 1**)**. The median age of participants at enrolment was 30 years (IQR 25–34 years) and the median gestational age was 18 weeks (IQR 13–24 weeks) (Table 1). At enrolment, half of the women (49%) reported being married or cohabitating with a partner and a third (34%) were formally employed. There were no differences in characteristics of women at enrolment between the Tshwane district and Cape Town cohorts as shown in supplementary material (Table S1).

**Table 1:** Baseline characteristics of mothers by HIV status at 1st ANC visit and STI prevalence during pregnancy in South Africa (2016–2019)

	All (n, %)	Women living without HIV	Women living with HIV	
Total	619	133 (21)	486 (79)	***p***-value
**Sociodemographic characteristics**				
Maternal age (median, IQR) years	**30 (25–34)**	26 (23–32)	30 (26–34)	< 0.001
GA at booking (median, IQR) weeks	**18 (13–24)**	19 (13–24)	18 (13–23)	0.78
Gravidity (median, IQR)	**3 (2–4)**	**2 (1–3)**	**3 (2–4)**	< 0.001
Parity (median, IQR)	**1 (1–2)**	**1 (0–2)**	**2 (1–2)**	< 0.001
**Education level completed**				
Primary	**220 (36)**	3 (2)	217 (45)	< 0.001
Secondary	**368 (59)**	121 (91)	247 (51)	
Tertiary (College/University)	**31 (5)**	9 (7)	22 (4)	
**Relationship with father of child**				
Married/Cohabiting	**305 (49)**	65 (49)	240 (49)	0.91
Not married/ Non-cohabiting	**289 (47)**	66 (49)	223 (46)	
No relationship	**25 (4)**	2 (2)	23 (5)	
**Employment status**				
Formal employment	**211 (34)**	36 (27)	175 (36)	0.06
Informal employment	**18 (3)**	2 (2)	16 (3)	
Unemployed/attending school	**390 (63)**	95 (71)	295 (61)	
**Site**				
Tshwane District	**380 (61)**	0 (0)	380 (78)	–
Cape Town	**239 (39)**	133 (100)	106 (22)	
**Clinical characteristic in this pregnancy**				
Any STI during pregnancy or postpartum				
Yes	**249 (40)**	40 (30)	209 (43)	0.007
No	**370 (60)**	93 (70)	277 (57)	
Any STI at 1st ANC visit				
Yes	**228 (37)**	36 (27)	192 (40)	0.008
No	**391 (63)**	97 (72)	294 (60)	
Any STI at 1st postnatal visit (**n**^**a**^ **= 571**)				
Yes	**45 (8)**	4 (3)	45 (9)	0.03
No	**526 (92)**	120 (97)	441 (91)	
Any STI at 1st ANC and 1st postnatal visit				
Yes	**30 (5)**	1 (1)	29 (6)	0.01
No	**589 (95)**	132 (99)	457 (94)	
*Chlamydia trachomatis* at 1st ANC				
Yes	**158 (26)**	27 (20)	131 (27)	0.11
No	**461 (74)**	106 (80)	355 (73)	
Neisseria gonorrhoea at 1st ANC				
Yes	**35 (6)**	3 (2)	32 (7)	0.05
No	**584 (94)**	130 (98)	454 (93)	
Trichomonas vaginalis				
Yes	**114 (18)**	14 (11)	100 (21)	0.008
No	**505 (82)**	119 (89)	386 (79)	
^b^Coinfections of CT, NG or TV at 1st ANC				
Yes	**72 (12)**	8 (6)	64 (13)	0.02
No	**547 (88)**	125 (82)	422 (87)	
**Sexual behaviour during pregnancy**				
Vaginal sex	**551 (89)**	126 (94)	425 (87)	0.01
Oral sex	**36 (6)**	6 (5)	30 (6)	0.46
Anal sex	**14 (2)**	3 (2)	11 (2)	0.99
2+ sex partners in the past 3 months	**18 (3)**	1 (1)	17 (4)	0.09
**Suspect partner of having another sex partner**				
No	**284 (46)**	77 (58)	201 (43)	0.02
Yes	**212 (34)**	35 (36)	177 (38)	
Don’t know	**117 (19)**	20 (15)	97 (20)	
N/A^c^	**6 (1)**	1 (1)	5 (1)	
**Partner’s serostatus**				
Concordant HIV status	**216 (35)**	92 (69)	124 (26)	< 0.001
Serodiscordant	**92 (15)**	4 (3)	88 (18)	
Serostatus unknown	**311 (50)**	37 (28)	274 (56)	

*IQR* Interquartile range, *n* number of participants, *GA* gestational age, *ANC* antenatal care

^a^number of women at 1st postnatal visit excluding pregnancy losses

^b^Co-infection with at least two infections of *Chlamydia trachomatis*, *Neisseria gonorrhoeae* or *Trichomonas vaginalis*

^c^Refers to women who didn’t have a partner, they were not in a relationship

### Prevalence of curable STI at first antenatal visit

At first ANC visit, 37% (228); 95% CI 33–41% were diagnosed with at least one STI. When stratified by HIV status, prevalence of any STI was 40% (192) in women living with HIV versus 27% (36) in women living without HIV. The most common infection at enrolment was *C. trachomatis* at 26% (158), with the infection more prevalent among women living with HIV, 27% (131) versus 20% (20) in women living without HIV, (*p*-value = 0.11), followed by *T. vaginalis* at 18% (120), with more infections among women living with HIV, 21% (106) versus 11% (14) in women living without HIV, (*p*-value = 0.008). *N. gonorrhoeae* had the lowest prevalence at enrolment with 6% (40) infected and 8% (37) in women living with HIV versus 2% (3) in women living without HIV, (*p*-value = 0.05). At enrolment, 12% (72) women had at least two infections of curable STIs, 13% (64) in women living with HIV versus 6% (8) in women living without HIV, (*p*-value = 0.02) (Table 1). At first postnatal visit, 8% (45) among women who had live births (*n* = 571) were diagnosed with at least one STI, 4% (21) were incident infection and 4% (24) were persistent infection. When stratified by HIV status, prevalence of any STI at first postnatal visit was 9% (45) in women living with HIV versus 3% (4) in women living without HIV. We compared the prevalence of at least one STIs in women living with HIV from Tshwane district 40% (151) versus Cape Town 39% (41). The prevalence for each STI was: *C. trachomatis* 29% (110) vs 20% (21), *T. vaginalis* 20% (77) vs 22% (23) and *N. gonorrhoeae* 6% (21) vs 10% (11) (Table S1). Most women (94%) were given their STI tests results before leaving the clinic and those who had a positive diagnosis received treatment on the same day in accordance with South African national guidelines.

### Pregnancy and birth outcomes

Among 619 women included for this analysis (Fig. 1), 93% (574) had singleton live births with a median birthweight of 3125 g (IQR 2780–3420). Of those in this analysis, 24% (146) of the women had an adverse pregnancy outcome making up the primary outcome of interest, the composite adverse outcome 25% (123) in women living with HIV and 17% (23) in women living without HIV. Overall, 5% (29) experienced a miscarriage and 2% (16) experienced a stillbirth. Among the live birth, 1% (3) were early neonatal deaths, 10% (62) delivered a preterm baby and 7% (35) with low birth weight among the full term babies (Table 2). There was no difference observed for pregnancy losses (miscarriage and stillbirth) between women with and without STI during pregnancy by HIV status, 6% (2) vs 6% (6) in women living without HIV and 7%(13) vs 8%(24) in women living with HIV. In women who had a live birth, the preterm delivery rates were higher, but the difference is not significant, among women diagnosed with a STI during pregnancy compared to those without a STI in both women living with HIV (12% (24) vs. 10% (29)) and women living without HIV (11% (4) vs. 5% (5)). There was no difference detected for low birthweight between women with and without a curable STI during pregnancy in both women living with and without HIV (RR; 1.00, 95% CI: 0.84–1.19 and RR; 1.02, 95% CI: 0.69–1.44 respectively).

**Table 2:** Adverse pregnancy and birth outcomes among women with and without STI at 1st ANC visit by HIV status in South Africa (2016–2019)

	All women	Women living without HIV - 133 (21)			Women living with HIV - 486 (79)		
	(***n*** = 619)	Any STI, n (%)	No STI, n (%)		Any STI, n (%)	No STI, n (%)	
Total of all women with pregnancy outcomes	n (%)	36 (27)	97 (73)	RR^**a**^(95% CI)	192 (40)	294 (60)	RR^**a**^(95% CI)
**Pregnancy losses**							
**Total pregnancy losses**	**45 (7)**	**2 (6)**	**6 (6)**		**13 (7)**	**24 (8)**	
Miscarriage GA ≤ 20 weeks	29 (5)	0 (0)	3 (3)	–	9 (5)	17 (6)	0.83 (0.36–1.91)
Stillbirth GA > 20 weeks	16 (2)	2 (6)	3 (3)	1.88 (0.30–11.85)	4 (2)	7 (2)	0.90 (0.25–3.12)
**Live birth outcomes**							
**Total live births**	**574 (93)**	**34 (95)**	**91 (94)**		**179 (93)**	**270 (92)**	
Full term delivery GA ≥ 37 weeks	509 (82)	30 (83)	85 (88)	ref	153 (80)	241 (82)	ref
Preterm delivery GA < 37 weeks	62 (10)	4 (11)	5 (5)	2.26 (0.57–9.00)	24 (12)	29 (10)	1.30 (0.73–2.32)
Early neonatal death	3 (1)	0 (0)	1 (1)	–	2 (1)	0 (0)	–
LBW < 2500 g	35 (7)	2 (7)	3 (4)	1.02 (0.69–1.44)	13 (9)	17 (7)	1.00 (0.84–1.19)
≥ 2500	473 (93)	28 (93)	82 (96)	ref	140 (91)	223 (93)	ref
Birth weight (median, IQR) grams (**n**^**b**^ **= 508**)	3175 (2880–3450)	3195 (3060–3750)	3360 (3020–3570)		3100 (2830–3370)	3175 (2855–3405)	
**Composite adverse outcome**							
Adverse outcome	146 (24)	8 (22)	15 (15)	1.43 (0.60–3.38)	52 (27)	71 (24)	1.12 (0.78–1.60)
No adverse outcome	473 (76)	28 (78)	82 (85)	ref	140 (73)	223 (76)	ref

*IQR* Interquartile range, *LBW* low birthweight, *GA* gestational age, *ANC* antenatal clinic, *CI* confidence interval, *RR* relative risk

Any STI refers to a positive test for *C. trachomatis, N.gonorrhoea* or *T.vaginalis* at first antenatal visit

^a^Calculated relative risk adjusting for maternal age, gestational age at enrolment, employment, marital status and gravidity

^b^ *n* = 508 which are infants born full term (excluding preterm babies)

Composite adverse outcome include all adverse pregnancy events: miscarriage, stillbirth, preterm birth, neonatal death, and low birthweight

In the modified Poisson regression with robust error measurements, STI diagnosis and treatment at the first ANC visit was not associated with a composite adverse outcome in women living with HIV and women living without HIV, adjusted relative risk (aRR); 1.43, 95% CI: 0.95–2.16 and 2.11, 95% CI: 0.89–5.01, respectively, adjusting for maternal age, gestational age at enrolment, employment, marital status and gravidity (Table 3). When restricting the analysis to *C. trachomatis* or *N. gonorrhoeae* at first ANC visit we found an association with adverse pregnancy outcome in women living with HIV (aRR; 1.57, 95% CI: 1.04–2.39). When stratified by each STI, *C. trachomatis* and *N. gonorrhoeae* diagnosis and treatment at first ANC, each were significantly associated with the composite adverse outcomes in women living with HIV ((aRR; 1.57, 95% CI: 1.04–2.39) and (aRR; 1.69, 95% CI: 1.09–3.08), respectively) (Table 3). STI diagnosis at first postnatal visit (a proxy of STI positivity at time of delivery) did not elevate the risk of an adverse birth outcome in women living with HIV or in women without HIV who had live birth and returned for postnatal visit (aRR; 0.90, 95% CI: 0.44–1.82 and aRR; 0.90, 95% CI: 0.45–1.79). In the sensitivity analysis, we excluded miscarriage in the composite adverve outcome, and having diagnosis with a curable STI at first ANC did not increase the risk of having a composite adverse outcome in women living with and without HIV (Table 3).

**Table 3:** Association between curable STI diagnosis at 1st ANC visit and adverse pregnancy outcomes among women living with and without HIV in South Africa (2016–2019)

	All women		Women living without HIV (*n* = 133)		Women living with HIV (*n* = 486)	
	^**a**^**Composite adverse outcome**		^**a**^**Composite adverse outcome**		^**a**^**Composite adverse outcome**	
	**RR (95% CI)**	**aRR (95% CI)**	**RR (95% CI)**	**aRR (95% CI)**	**RR (95% CI)**	**aRR (95% CI)**
^c^Any STI at 1st ANC	1.19 (0.89–1.59)	1.26 (0.94–1.68)	1.43 (0.66–3.10)	2.11 (0.89–5.01)	1.12 (0.82–1.52)	1.43 (0.95–2.16)
^d^Any STI at 1st ANC (*C.trachomatis/N.gonorrhoeae*)	1.15 (0.85–1.57)	1.24 (0.91–1.68)	1.32 (0.57–3.05)	1.96 (0.72–5.31)	1.10 (0.79–1.53)	**1.57 (1.03–2.38)**
*C. trachomatis* during pregnancy	1.13 (0.83–1.55)	1.22 (0.89–1.67)	1.38 (0.60–3.18)	2.01 (0.75–5.33)	1.07 (0.76–1.50)	**1.57 (1.04–2.39)**
*T. vaginalis* during pregnancy	1.09 (0.77–1.56)	1.11 (0.78–1.58)	1.27 (0.43–3.76)	1.73 (0.56–5.26)	1.03 (0.71–1.50)	1.06 (0.65–1.74)
*N. gonorrhoeae* during pregnancy	1.35 (0.81–2.26)	1.48 (0.89–2.44)	1.96 (0.37–10.26)	3.58 (0.58–21.84)	1.25 (0.73–2.15)	**1.69 (1.09–3.08)**
Coinfection of STIs at 1st ANC	1.06 (0.69–1.63)	1.17 (0.76–1.79)	1.48 (0.41–5.28)	2.96 (0.68–12.80)	0.98 (0.62–1.55)	1.45 (0.86–2.46)
	^**b**^**Composite adverse outcome**		^**b**^**Composite adverse outcome**		^**b**^**Composite adverse outcome**	
	**RR (95% CI)**	**aRR (95% CI)**	**RR (95% CI)**	**aRR (95% CI)**	**RR (95% CI)**	**aRR (95% CI)**
^c^Any STI at 1st ANC	1.30 (0.94–1.81)	1.34 (0.96–1.87)	1.74 (0.77–3.91)	2.53 (0.99–6.43)	1.20 (0.84–1.71)	1.31 (0.85–2.01)
^d^Any STI at 1st ANC (*C. trachomatis/N. gonorrhoeae*)	1.23 (0.87–1.74)	1.28 (0.89–1.82)	1.56 (0.65–3.70)	2.20 (0.76–6.35)	1.15 (0.79–1.68)	1.45 (0.92–2.27)
*C. trachomatis* during pregnancy	1.19 (0.83–169)	1.24 (0.86–1.79)	1.63 (0.69–3.86)	2.27 (0.81–6.35)	1.09 (0.74–1.62)	1.43 (0.91–2.25)
*T. vaginalis* during pregnancy	1.18 (0.80–1.75)	1.18 (0.79–1.75)	1.46 (0.48–4.38)	1.94 (0.61–6.41)	1.11 (0.73–1.69)	1.02 (0.60–1.73)
*N. gonorrhoeae* during pregnancy	1.52 (0.88–2.64)	1.59 (0.93–2.72)	2.22 (0.42–11.71)	3.37 (0.54–20.79)	1.41 (0.79–2.53)	1.62 (0.85–3.11)
Coinfection of STIs at 1st ANC	1.17 (0.73–1.87)	1.22 (0.76–1.96)	1.69 (0.47–6.08)	2.95 (0.68–12.73)	1.08 (0.65–1.78)	1.45 (0.83–2.54)
^e^Any STI at 1st postnatal visit	0.89 (0.44–1.82)	0.90 (0.45–1.80)	–	–	0.90 (0.44–1.82)	0.90 (0.45–1.79)

*RR* relative risk, *aRR* adjusted relative risk, *STI* sexually transmitted infection, *CI* confidence interval, *ANC* antenatal care

Modified Poisson regression with robust error measurements was used to calculate RR and aRR adjusting for maternal age, gestational age at enrolment, employment, marital status, gravidity and CD4 count at enrolment for women living with HIV

STIs refers to *C. trachomatis, T. vaginalis* and *N. gonorrhoeae*

^a^Composite adverse outcome combines low birthweight< 2500 g, preterm birth, miscarriage, stillbirth and early neonatal death

^b^Composite adverse outcome combines low birthweight< 2500 g, preterm birth, stillbirth and early neonatal death, excluding miscarriage

^c^Any STI at 1st ANC is the exposure of interest

^d^Any STI at 1st ANC excluding *T. vaginalis* is the exposure of interest

^e^Any STI at 1st postnatal visit excluding women who experienced pregnancy losses

## Discussion

This prospective observational study implemented aetiological STI testing and treatment for the most common STIs (*C. trachomatis, N. gonorrhoeae* and *T. vaginalis*) as part of ANC at community health centres in two districts (Tshwane and Cape Town) in South Africa. The study shows high prevalence of curable STIs at enrolment of 37% in pregnant women living with and without HIV. STI prevalence was 1.48 times higher in women living with HIV versus women living without HIV. We observed a 78% reduction in prevalence of STIs at postnatal visit, (from 37 to 8%), which we used as proxy for STI status at time of delivery with only half of the infections incident [26].

In our study, we did not find an association between STI diagnosis and treatment at first ANC visit and composite adverse pregnancy outcome in women living with and without HIV. However, when we stratified the analysis by each STI, *C. trachomatis* and *N. gonorrhoeae* were each significantly associated with adverse pregnancy and birth outcomes among women living with HIV with a 1.57 and 1.69 fold increased risk respectively. Despite diagnosis and treatment fairly early in pregnancy, the association of STI diagnosis and composite adverse pregnancy outcome was present for *C. trachomatis* and *N. gonorrhoeae* among women living with HIV but not women living without HIV. When we excluded miscarriage from the composite adverse outcome, there was no difference in the risk of adverse pregnancy and birth outcomes among women living with and without HIV.

In our study, most women (94%) were given their STI tests results before leaving the clinic and those who had a positive diagnosis received treatment on the same day [32]. The overall finding of no association between STI diagnosis at first ANC visit and composite adverse pregnancy outcome could be a result of timely diagnosis and treatment of STIs at first ANC visit. The study provided partner referral for STI treatment, but it is not known how many men were treated for STIs. Hence, 8% of women had a STI in their postpartum visit which is a proxy for infection at delivery. More work is needed to improve partner expedited therapy and treatment, especially in pregnancy [36]. It is well documented that untreated STIs during pregnancy are associated with adverse pregnancy outcomes [1, 4, 11, 12]. However, the association found between a diagnosis with *C. trachomatis* and *N. gonorrhoeae* at first ANC visit with adverse pregnancy and birth outcomes among women living with HIV could be highlighting that there is complex interactions in STIs, HIV and pregnancy outcomes. Importantly, implementation of aetiological testing and treating of curable STIs at the first ANC visit resulted in reducation of the burden of STIs at the time of delivery. Although the optimal timing of STI management to improve pregnancy outcomes is unknown, reducing STI burden during pregnancy is likely beneficial in effort to improve maternal health during pregnancy [1, 4, 8].

In the stratified analysis by each STI, our finding of *C. trachomatis* and *N. gonorrhoeae* at first ANC visit associated with increased risk of composite adverse outcome is consistent with previous studies that have assessed this relationship [1, 5, 6, 11]. These associations were present after adjusting for maternal age, gestational age at enrolment, employment, marital status and gravidity. However, all women living with HIV in this study were on antiretroviral therapy (ART) (predominately first line efavirenz-based regimen) either prior to conception or initiated during pregnancy. It is possible that HIV and ART could have amplified the risk of having an adverse outcome as a result of in utero exposure of the foetus to HIV and or ARVs [27–31]. Nevertheless, the association of *N. gonorrhoeae* infection during pregnancy and low birthweight is biologically plausible as *N. gonorrhoeae* can cross the placenta, impact placental and fetal development leading to intrauterine growth restriction, which subsequently results in low birthweight [37]. *C. trachomatis* infection during pregnancy may infect the placenta, which may lead to preterm delivery [7, 8]. There are many possible explanations for discrepancies in findings between published studies, some showing association [1, 4–8, 12] and others showing no association [11, 38, 39]. Heterogeneity in studies could be due to small numbers of adverse events in underpowered studies, time of detection and treatment of the infection or inconsistency in the types of tests used for screening of STIs. The impact of STIs at different duration of pregnancy is unknown and it is not clear whether a STI in early pregnancy impacts outcomes later. *C. trachomatis* is associated with upper genital tract infections that result in intrauterine colonization that can occur early in gestation or preconception [40]. Therefore, timing of screening and treatment of curable STIs is an important factor to be considered when evaluating the association.

In the sensitivity analysis, we excluded miscarriage in the composite adverve outcome as the estimate of miscarriage is usually underestimated due to left truncation as women attend first ANC visit late in gestation. However, we did not find any association between STI at first ANC and adverse outcomes when we included or excluded the underestimate outcome of miscarriage in the composite adverse outcome.

This study provides important findings to suggest that universal testing of STIs at first ANC visit using diagnostic tests may be justified in routine ANC especially for pregnant women living with HIV, given the high prevalence of antenatal HIV in South Africa [25]. The integration of STI screening in routine ANC could also help reduce the risk of HIV acquisition and might help eliminate vertical HIV transmission [11].

However, the study is not without limitations. Our study over-represented women living with HIV relative to women without HIV, as the study in Tshwane District enrolled women living with HIV only. However, collating data from two geographically distinct areas increased generalizability of our study findings in South African township settings. We included an underestimate of miscarriage outcomes in our composite outcome of interest, acknowledging the limitation that most women attend for their first antenatal visit late in gestation after the typical occurrence period of miscarriage. Due to missing infant sex in our dataset, we were limited from using standardized birthweight adjusted for infant sex and gestational age to evaluate infants who were small for gestational age. Unfortunately, we could not evaluate small-for-gestational age infants as part of the composite adverse outcome. Many women were infected with STIs at first ANC and subsequent visits. However, we were not able to ascertain if women were re-infected or if it was a failure to cure. In this study, we tested for the three most common STIs (*C. trachomatis, T. vaginalis* and *N. gonorrhoeae*), but infections may co-exist and could have masked our findings. Therefore, unobserved confounding should be taken into account when interpreting our findings. Lastly, we used STI test results at postnatal visit as proxy for STI status at time of delivery which could have been biased by post-delivery selection bias whereby all women who experienced adverse pregnancy events did not return for postnatal follow up. Also, a 4 weeks window period was given for the first postnatal visit, which could have allowed for post delivery incident infection if women had resumed sexual intercourse, biasing our STI prevalence estimate at time of delivery.

## Conclusion

Detecting and treating of STIs at first ANC were not associated with increased risk of having a composite adverse pregnancy outcome overall. However, in women living with HIV infection, a positive diagnosis with *C. trachomatis* or *N. gonorrhoeae* had an association with composite adverse outcome independently. Our overall finding suggest that aetiological STI testing and treating in routine ANC can provide an effective intervention urgently needed to reduce the high burden of STIs in pregnant women in South Africa, and possibly improve pregnancy outcomes. The interaction between STIs, HIV and pregnancy outcomes is complex and requires studies designed to identify clinical significance of these interations, especially in South African setting with high double burden of HIV and STIs.

## Supplementary Information

**Additional file 1.**

## Data Availability

The datasets used and analysed during this current study are available from corresponding author on request.

## Abbreviations

ANC: Antenatal clinic
ARV: Antiretroviral
ART: Antiretroviral therapy
GA: Gestational age
STIs: Sexually transmitted infections
